# Chloroform extract and acetyl-11-keto-beta-boswellic acid from *Boswellia dalzielii* stem bark induce apoptosis and cell cycle blockage in AW8507 cells

**DOI:** 10.1186/s43046-021-00075-3

**Published:** 2021-08-09

**Authors:** Akinbobola Peace Otitoju, Ishaya Yohanna Longdet, Taiwo Emmanuel Alemika, Vikram Prakash Gota

**Affiliations:** 1 Department of Biochemistry, Faculty of Basic Medical Sciences, University of Jos, Jos, Nigeria; 2 Advanced Centre for Treatment Research and Education in Cancer (ACTREC), Department of Clinical Pharmacology, Tata Memorial Centre, Kharghar, Navi, Mumbai 410210 India; 3 Medical Biotechnology Department, National Biotechnology Development Agency, Abuja, Nigeria; 4 Department of Pharmaceutical Chemistry, Faculty of Pharmaceutical Science, University of Jos, Jos, Nigeria

**Keywords:** *Boswellia dalzielii*; HPLC–MS, Reverse virtual screening, Acetyl-11-keto-beta-boswellic acid

## Abstract

**Background:**

Globally, head and neck cancer is the sixth most common cancer. Despite the advancement in treatment, drug resistance remains a major cause for setback. In an earlier work, the authors reported that *Boswellia dalzielii* (Hutch) stem bark exhibited dose-dependent cytotoxicity in head and neck cancer cells, AW8507. Therefore, the cell death induction effect of *Boswellia dalzielii* stem bark chloroform extract in head and neck cancer cell line, AW8507, and its derived constituent on cell cycle and apoptosis proteins was further investigated.

**Methods:**

The cell death induction activity of the *Boswellia dalzielii* stem bark chloroform fraction (CLBD) in AW8507 was determined using Annexin V-FITC/PI staining in flow cytometry. High-performance liquid chromatography-mass spectrometry was employed for compounds analysis of the CLBD, and reverse virtual screening was used to identify the mechanism of action of the compound, acetyl-11-keto-beta-boswellic acid, that was elucidated in the *Boswellia dalzielii* chloroform fraction.

**Results:**

The data obtained showed that *Boswellia dalzielii* stem bark Chloroform extract increased the percentage of cells presenting for early apoptosis from 4.14 to 10.10% in AW8507 cells. High-performance liquid chromatography-mass spectrometry analysis of the chloroform fraction identified acetyl-11-keto-beta-boswellic acid. Reverse virtual screening on selected proteins showed that acetyl-11-keto-beta-boswellic acid is a multi-protein target compound. It binds preferably to phosphorylated-cyclin dependent kinase 1 (p-CDK1) (binding score =  − 9.2 kcal/mol), blocking the activation of cyclin B-CDK1 needed for cell cycle progression at G2/M phase of the cell cycle. Acetyl-11-keto-beta-boswellic acid also binds more tightly with αβ tubulin (binding score = 8.9 kcal/mol) than with the standard drug, docetaxel (binding score = 8.3 kcal/mol).

**Conclusions:**

The results obtained confirmed the culpability of *Boswellia dalzielii*-derived acetyl-11-keto-beta-boswellic acid in the obstruction of the cell cycle progression in head and neck cancer cell line, AW8507; and the induction of apoptosis earlier reported for *Boswellia dalzielii* (Hutch) stem bark. Additional in vitro and/or in vivo studies would be required to validate in silico observations.

## Background

Despite the growing understanding of tumor biology and advancement in the treatment of head and neck cancer, morbidity and mortality rates have not abated due mainly to drug resistance among several factors. There are over 600,000 incidences and 350,000 deaths associated with head and neck cancer globally [1]. The public health burden of head and neck squamous cell carcinoma is profound and the incidence is expected to rise by 30% by the year 2030 [2]. Studies have established that cancer can be considered as a disease of the cell cycle due to ineffective cell cycle checkpoint control [3]. Cell cycle arrest of cancer cells is therefore regarded as one of the target mechanisms in cancer treatment [3]. Likewise, apoptosis, a significant terminal pathway for cells of multicellular organisms, that causes a diversity of biological events such as proliferation/homeostasis, differentiation, development and elimination of harmful cells as a protective strategy to remove diseased cells [4], represents one of the prominent mechanisms of chemotherapy [5]. Many natural compounds (curcumin, resveratrol, epigallocatechin-3-gallate, and quercetin) from plants have been found to inhibit one or more pathways that contribute to proliferation [6]. *Boswellia dalzielii* is the West African species of the frankincense genus and its phytochemical screening revealed the presence of saponins, tannins, flavonoids, cardiac glycosides, steroids, and terpenes [7]. The chemical components of *B. dalzielii* gum resin have been reported to contain beta-sistosterol, resveratrol, garlic acid, incensole, and protocatechuic acid [8]. The cytotoxic activity of *Boswellia dalzielii* gum resin and leaves were previously investigated on brine shrimp and ovarian cancer cells, respectively [9, 10]. In an earlier work, the authors reported that *Boswellia dalzielii* stem bark exhibited dose-dependent cytotoxic activity in head and neck squamous cell carcinoma (AW8507 cell line) and blocked the cell cycle progression at G2/M [11]. Thus, the aim of this study was to evaluate the cell death induction activity of the chloroform extract in AW8507 cells, determine the active constituent and its activity against selected enzymes in silico.

## Methods

### *Boswellia dalzielii *stem bark

*Boswellia dalzielii* stem bark was gathered at Dutsen Hanwa area of Zaria (latitude 11° 06′ 40.61″ N, longitude 7° 43′ 21.72″ E), Kaduna state, Northern Nigeria, in the month of October, 2017, and was authenticated at Biological Sciences Department, Ahmadu Bello University, Zaria, and a voucher specimen was deposited with voucher number 900121.

### Extraction, fractionation, and phytochemical identification

**Fig. 1:**
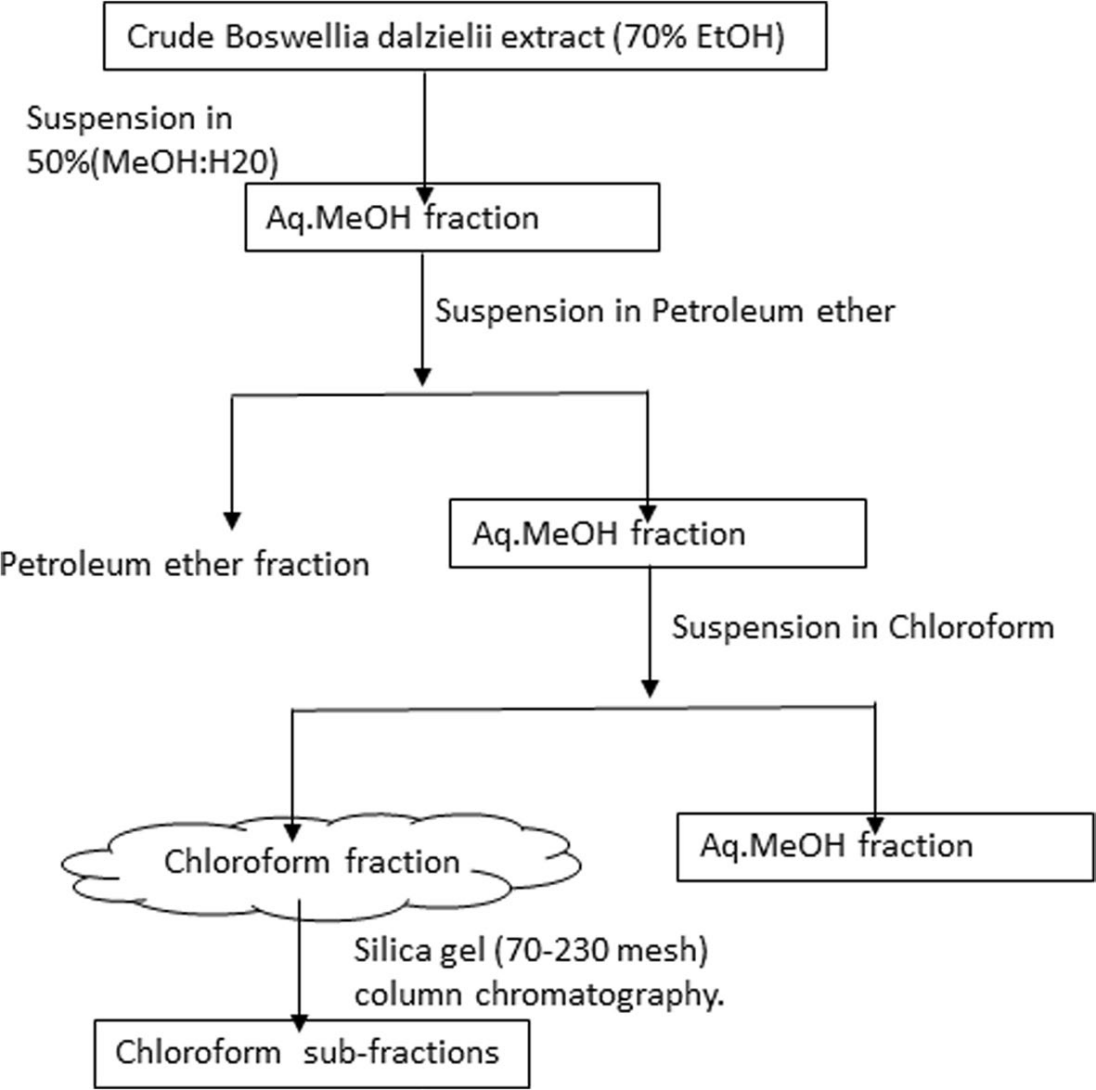
Extraction and fractionation of *Boswellia dalzielii* stem bark

The chloroform fraction of *Boswellia dalzielii* stem bark was prepared as earlier described [11]. Further, the chloroform fraction was subjected to silica gel (70–230 mesh) column chromatography. Briefly, silica gel was mixed with n-hexane to form a homogenous suspension/slurry and stirred using a glass-stirring rod to remove bubbles. The silica gel slurry was then poured into a glass column. The sample to load on the column was prepared by dissolving 3.7 g of the chloroform fraction in 50 ml of hexane. A preparative thin layer chromatography (TLC) had been carried out in which the chloroform fraction were separated in a mobile phase of chloroform/methanol (99:1). To the solution, 10 g of silica was added and mixed by stirring with a glass rod. The mixture was allowed to dry at room temperature. The dried silica/*Boswellia dalzielii* fraction mixture was layered on the column layer bed. The column was first eluted with n-hexane as the mobile phase with the polarity increasing by 5% increments of chloroform. After getting to 100% chloroform, the polarity was further increased by 5% increments of methanol (Fig. 1). Six sub-fractions were collected in glass beakers. The collected fractions were concentrated to dryness at room temperature.

### Cell culture

The head and neck cancer cell line (AW8507) was generously obtained from the Advanced Centre for Treatment Research and Education in Cancer (ACTREC), Tata Memorial Centre, Kharghar, Navi-Mumbai, 410,210, India, and were cultured in Dulbecco’s minimum essential medium (DMEM) with 10% fetal bovine serum (FBS) and 50 μg/mL antibiotics. The cells were incubated at 37 °C in CO_2_ incubator in an atmosphere of humidified 5% CO_2_ and 95% air. The cells were maintained by sub-culturing in 25-cm^3^ tissue culture flasks. Cells growing in the exponential phase were used for cell death determination assay.

### Determination of cell death by Annexin V-propidium iodide (AnnV-PI)

Mode of cell death induced by the chloroform fraction of *Boswellia dalzielii* stem bark (CLBD) was evaluated by using Annexin V-FITC Apoptosis Detection Kit (BD Biosciences). Briefly, the cells of AW8507 (3 mL, 1 × 10^5^ cells/mL) were seeded into each well of 6-well plates and incubated for 24 h (cell attachment and recovery). After incubation (24 h), the untreated and treated (20 µg/mL) cells were harvested and washed with cold phosphate-buffered saline (PBS) solution. Subsequently, the cells were mixed with 190 µL of pre-diluted binding buffer (1 ×) containing Annexin Fluorescein 5-isothiocynate (Annexin V-FITC) (5 µL) and of propidium iodide (PI) (1 µL) (100 μg/mL) and further incubated for 15 min at 37 °C in the dark. Subsequently, 400 µL of binding buffer (1 ×) was added into each tube. The percentage of cell undergoing apoptosis and necrosis was quantified using a flow cytometer (Becton Dickinson Accuri, San Diego, CA, USA) equipped with Cell Quest software within 1 h.

### HPLC–MS analysis of chloroform extract sub-fractions

The sub-fractions obtained from the chloroform extract were subjected to further analysis by high-performance liquid chromatography and mass spectrometry. For the analysis, 0.5–1.0 mg/ml of CLBD sub-fractions was dissolved in methanol. After that, it was filtered through 0.45 µm membrane filter prior to the injection into HPLC–MS system. HPLC–MS system was Agilent 1260 infinity HPLC (UV Detector wavelength, 225 ± 50 nm bandwidth), auto-sampler (injection volume 1 µl), and thermostated column (Phenomenex Kinetex XB-C18, 50 × 4.6 mm, 2.6 µm) (40^∘^C). The mobile phase A was 99.9% water with 0.1% acetic acid, while mobile phase B was 99.9% acetonitrile with 0.1% acetic acid. The flow rate was 2 mL/min. Agilent 6130 single quadrupole mass spectrometer (scanning in ES + / − and APCI over 70–1100 m/z) and Agilent 1290 Infinity II Evaporative Light Scattering Detector (ELSD) was used for measuring mass spectra. Agilent Mass Hunter software was used for data acquisition and processing. Compounds in the chloroform extract sample were tentatively identified by comparison of their monoisotopic/exact masses, mass fingerprints, and reference literatures.

### Reverse virtual screening of Acety-11-keto-beta-boswellic acid on selected proteins of the cell cycle

**Fig. 2:**
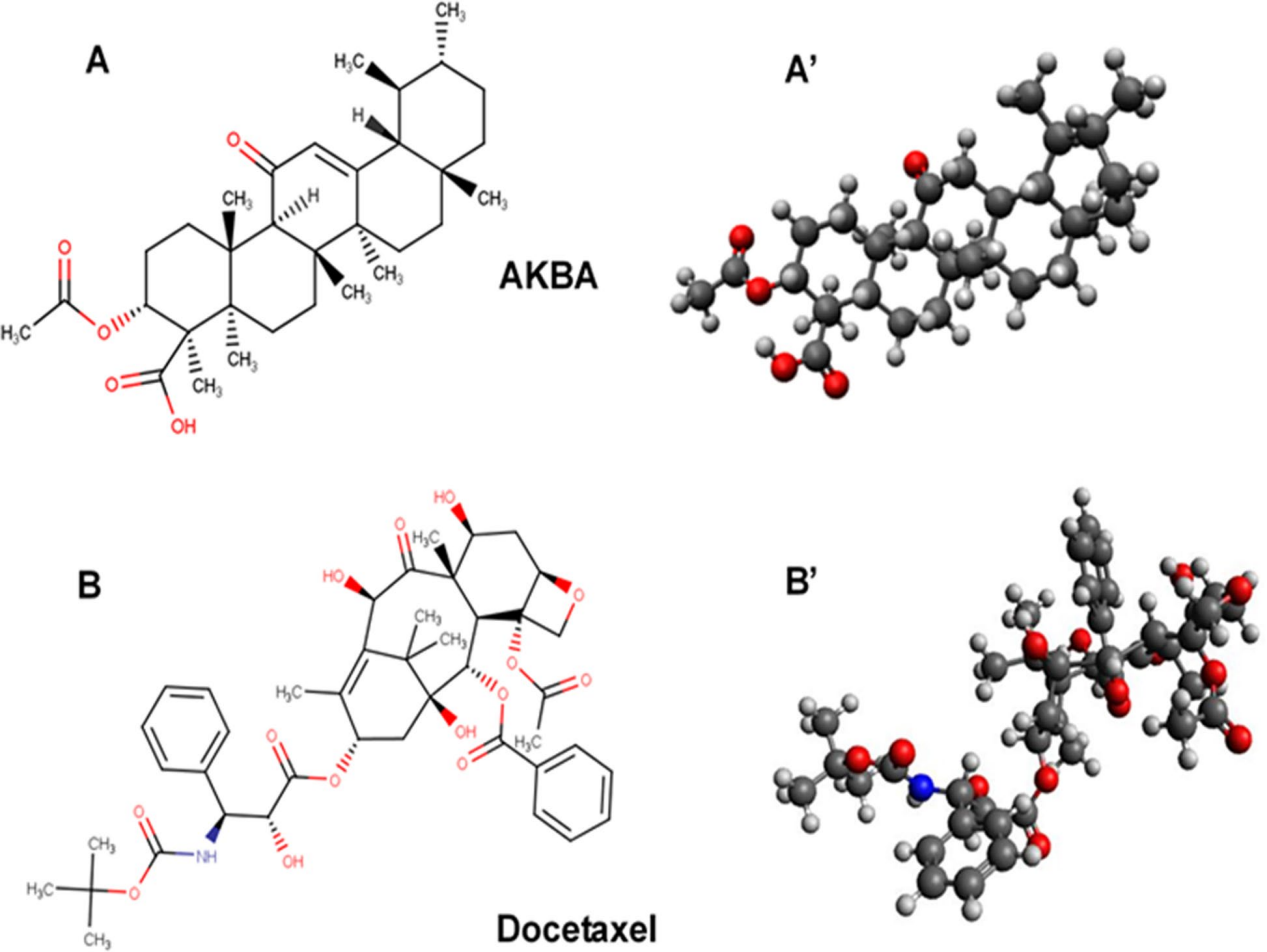
2D and optimized structures of the test compound acetyl-11-keto-beta-boswellic acid (AKBA) (**A**) and the standard compound, docetaxel (TXL) (**B**)

A reverse virtual screening (rVS) was performed to specifically determine the binding affinities of Acety-11-keto-beta-boswellic acid (AKBA) in the selected proteins [12]. Briefly, a total of 24 proteins (Forkhead box protein M1 (FOXM1), Aurora kinase, checkpoint kinase 1 (CHK1), serine/threonine polo-like kinase 1 (PLK1), Cell Division Cycle 25 homolog C (CDC25C), Cyclin B1, p-CDK1, DNA topoisomerase 2 alpha (TOP2A), phosphoinositide 3-kinase (PI3K), protein kinase B (Akt), proto-oncogene Neu (HER2), mesenchymal epithelia transition factor (c-MET), tumor protein p53 (TP53), mouse double minute 2 homolog protein (MDM2), Janus tyrosine kinase (JAK), signal transducer and activator of transcription 3 (STAT3), mitogen activated protein kinase (MAPK), vascular endothelia growth factor receptor (VEGFR), caspase 7, caspase 3, proto-oncogene tyrosine-protein kinase Src, B-Raf proto-oncogene serine/threonine-protein kinase, epidermal growth factor receptor (EGFR), and αβ tubulin) were selected to form a biomolecular library. The individual crystal structures of the proteins were retrieved from the protein data bank (PDB). The PDB entry values are 3G73^8^, 4BYJ^9^, 2C3K^10^, 2RKU^11^, 3OP3^12^, 6GU4^13^, 6GU4^13^, 5GWK^14^, 5JHB^15^, 3D0E^16^, 3PP0^17^, 4KNB^18^, 5O1C^19^, 5LN2^20^, 3KRR^21^, 6NUQ^22^, 5MTX^23^, 4AGD^24^, 1I51^25^, 1RHQ^26^, 3EL7^27^, 3C4C^28^, 5XDL^29^, and 1TUB^30^. The protein structures were individually prepared on the graphical user interface (GUI) of University of California at San Francisco (UCSF) chimera. The proteins were further prepared on the ADT interface where non-polar hydrogens and standard charges were added. Using coordinates from the co-crystallized ligands, target binding sites of the proteins were defined using a gridbox with size and center values that varied across the proteins. Each protein was then saved in.pdbqt formats to form the protein library against which the test compound was virtually screened. The 2-dimensional (2D) structure of the test compound, acetyl-11-keto-beta-boswellic acid, was prepared using the MarvinSketch graphical user interphase, after which it was structurally and geometrically optimized with a universal force field (UFF) using the Avogadro module [13]. Afterward, acetyl-11-keto-boswellic acid was prepared on AutoDock tools, torsions adjusted and rotatable/non-rotatable bonds were set. This was also saved in.pdbqt format. Reverse virtual screening was then performed using the AutoDock Vina software. Binding scores of acetyl-11-keto-beta-boswellic acid to each protein were then obtained. On the other hand, Docetaxel (TXL) was used as the standard molecule for comparatively measuring the binding activity of acetyl-11-keto-beta-boswellic acid (AKBA). To this effect, docetaxel was retrieved from the PubChem drug repository after which it was optimized similarly to AKBA (Fig. 2). Docetaxel (TXL) was docked singly to αβ Tubulin using AutoDock tools since docetaxel reportedly targets and bind the β domain of αβ Tubulin.

## Results

### *Boswellia dalzielii* stem bark induced apoptosis in head and neck squamous cell carcinoma (HNSCC)

**Fig. 3:**
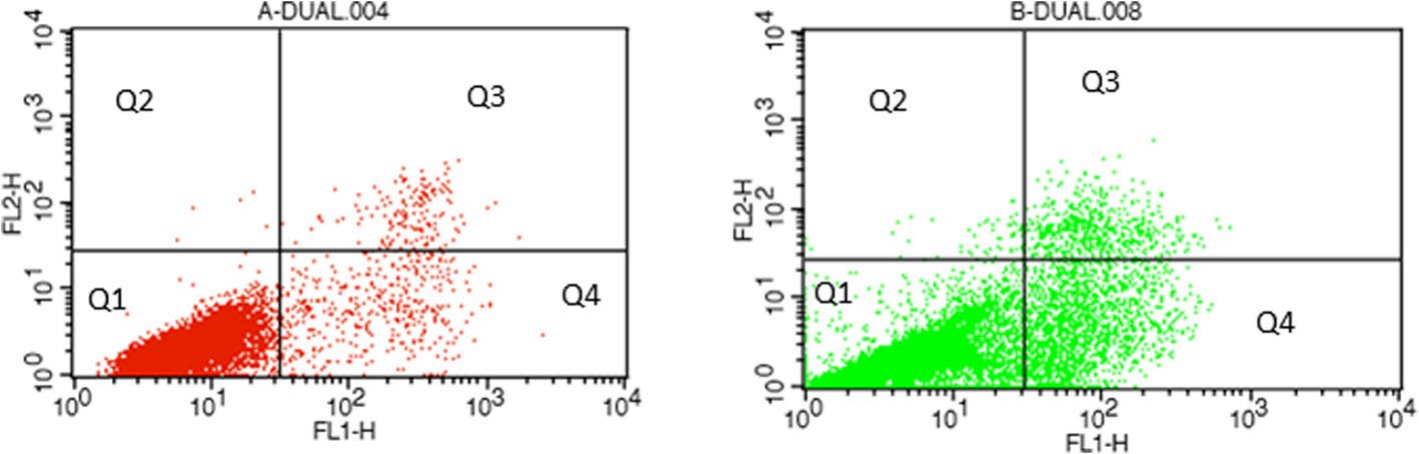
Cell death induction activity of *Boswellia dalzielii* stem bark chloroform extract in AW8507 cell. Viable cells (Q1), early apoptotic (Q4), late apoptotic (Q3), and dead (Q2) cells. A-DUAL, 004 (untreated AW8507 cells (control)); B-DUAL. 008 (chloroform extract treated AW8507 cells)

As shown in Fig. 3, there was an increase in the percentage of cells presenting for early apoptosis (Q4), from 4.14 to 10.10%, and a significant reduction in the percentage of viable cells from 94.20% in the untreated control cells to 86.42% observed in the CLBD treated cells (Q1). Similarly, percentage of cells presenting for the second and third quadrant (Q2 and Q3) the untreated control AW8507 cells to the CLBD treated cells, increased from 0.09% and 1.61% to 0.31% and 3.17% respectively.

### HPLC–MS analysis of *Boswellia dalzielii* stem bark chloroform fraction revealed the presence of acetyl-11-keto-beta-boswellic acid (AKBA)

**Fig. 4:**
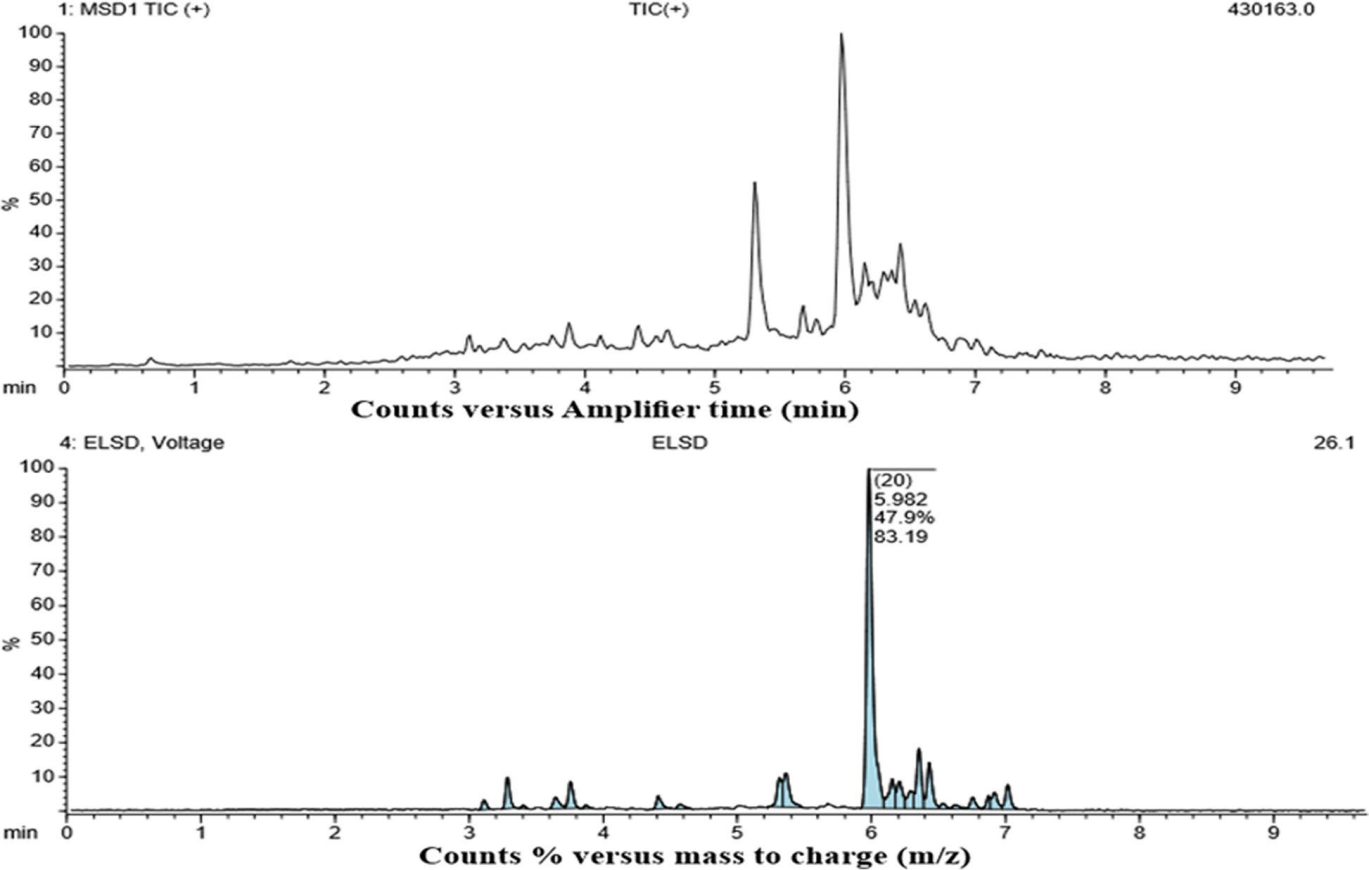
Total ion current (TIC) and extracted ion chromatograms of acetyl-11-keto-beta-boswellic acid (AKBA)

The HPLC–MS analysis of chloroform extract exhibited several compounds within the total ion chromatogram (TIC) (m/z 50–1000). The mass obtained from the ion chromatogram (± 2 ppm) of molecular ion (m/z 512.3) as shown in Fig. 4 represent the putative compound acetyl-11-keto-beta-boswellic acid. Acetyl-11-keto-beta-boswellic acid has a molecular mass of 512.7 g/mol and empirical formula, C_32_H_48_O_5._

### Reverse virtual screening of AKBA on selected proteins revealed it is a multi-protein target compound

**Fig. 5:**
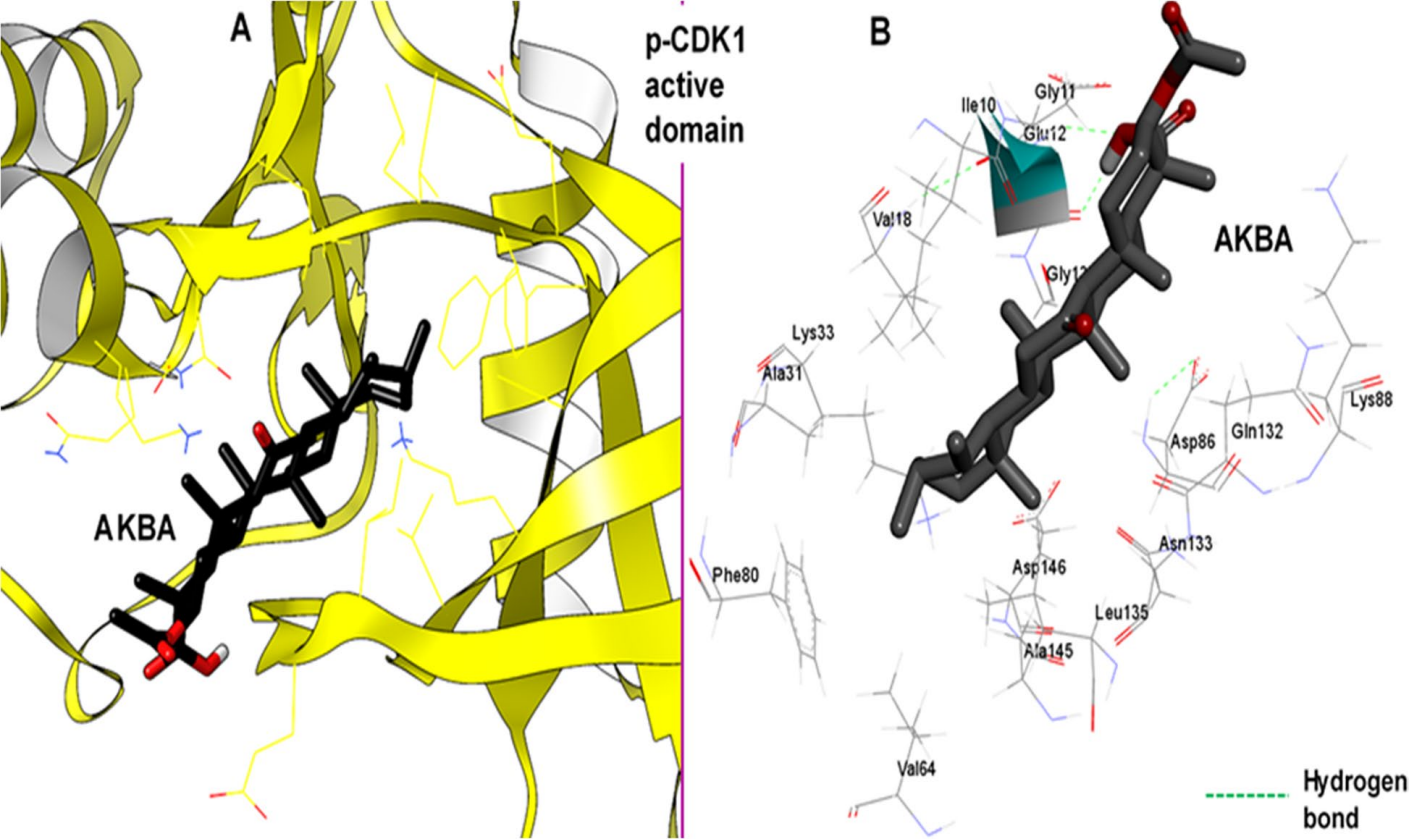
Interaction mechanisms of AKBA at the active domain of p-CDK1. **A** Binding orientation of AKBA at the target site. **B** Crucial residues that complementarily interact with AKBA at the p-CDK1 binding domain

Results of the reverse virtual screening presented in Table 1 showed that AKBA exhibited the most favorable affinity to p-CDK1 (binding score =  − 9.2 kcal/mol). As shown in Fig. 5, strong hydrogen bond were observed between AKBA and Glu12, Val18, and Asp86 at the crucial p-cdk1 binding site while the aromatic ring of Phe80 would allow for the formation of a stable π-π interaction with the proximal dimethyl-hexane ring. Alkyl groups of Lys33, Val18, Ala145, Leu135, Ile10, Ala31, and Val64 could as well form stable alkyl interactions and π-alkyl interactions with the aromatic rings in AKBA. The results in Table 1 also showed that AKBA is a multi-target compound as it binds to EGFR, B-Raf, Src kinase, MAPK, PI3K, and CHK1 (binding score =  − 8.6, − 8.8, − 8.7, − 8.9, − 8.3, and 8.5 kcal/mol respectively). Furthermore, Table 2 showed the distinct activities between AKBA and the standard drug, TXL, evaluated against αβ Tubulin; TXL had a lower binding affinity (binding score =  − 8.3 kcal/mol) as compared with AKBA (binding score =  − 8.9 kcal/mol).

**Table 1:** Reverse virtual screening analyses of AKBA against selected proteins

Target protein	PDB entry	Binding scores (kcal/mol)
FOXM1	3G73^8^	− 5.2
Aurora kinase	4BYJ^9^	− 7.9
CHK1	2C3K^10^	− 8.5
PLK1	2RKU^11^	− 7.7
CDC25C	3OP3^12^	+ 230.9
Cyclin B1	6GU4^13^	− 6.7
p-CDK1	6GU4^13^	− 9.2
TOP2A	5GWK^14^	− 6.3
PI3K	5JHB^15^	− 8.5
Akt	3D0E^16^	− 0.6
HER2	3PP0^17^	2.1
c-MET	4KNB^18^	− 7.9
TP53	5O1C^19^	− 5.2
MDM2	5LN2^20^	− 6.8
JAK	3KRR^21^	− 6.8
STAT3	6NUQ^22^	− 6.9
MAPK	5MTX^23^	− 8.9
VEGFR	4AGD^24^	− 6..6
Caspase 7	1I51^25^	− 7.3
Caspase 3	1RHQ^26^	− 7.4
Src	3EL7^27^	− 8.7
B-Raf	3C4C^28^	− 8.8
EGFR	5XDL^29^	− 8.6

**Table 2:** Reverse virtual screening of Acetyl-keto-11-beta-boswellic acid (AKBA) and docetaxel (TXL) against αβ Tubulin

Target protein	PDB entry	Ligand-protein complex	Binding scores (kcal/mol)
αβ Tubulin	1TUB^30^		AKBA (up) = -8.9
			TXL (down) = -8.3

In the structural depictions, AKBA (black) and TXL (red) occupy the defined active site of the target protein which is employed in its crystallographic state as retrieved from the protein data bank

In the structural depictions, AKBA (black) and TXL (red) occupy the defined active site of the target protein which is employed in its crystallographic state as retrieved from the protein data bank.

## Discussion

Over the years, many natural compounds extracted from different plants and presenting significant pharmacological effects have been used for the treatment of various chronic diseases. Currently, more than 10,000 phytochemicals made up of tannins, flavones, triterpenoids, steroids, saponins, and alkaloids have been identified, and many more are yet to be discovered [14]. As shown in Fig. 3, chloroform extract obtained from *Boswellia dalzielii* stem bark ethanol extract (CLBD) induced apoptosis in AW8507 cells. Apoptosis is understood to play a key role in the survival and proliferation of the neoplastic cells, failure to activate apoptosis represents as one of the major obstacles to the success of a particular cancer treatment [15]. Some of the compounds identified in CLBD (acetyl-11-keto-beta-boswellic acid, incensole, n-hexadecanoic acid, and decanoic acid) have been previously reported in various extracts of *Boswellia dalzielii* [11]. Acetyl-11-keto-beta-boswellic acid identified in the chloroform extract of the *Boswellia dalzielii* stem bark (CLBD) via the HPLC–MS technique is one of a series of pentacyclic molecules called the boswellic acids which derived from the *Boswellia* genus [16]. Reports suggest that acetyl-11-keto-beta-boswellic (AKBA) regulates a variety of molecular targets such as 5-lipoxygenase, nuclear factor-kappa B (NF-κB), cathelicidine peptide (LL-37), hypoxia inducible factor 1 (HIF-1), and other molecules that contribute to inflammation and tumor progression [17, 18].

**Fig. 6:**
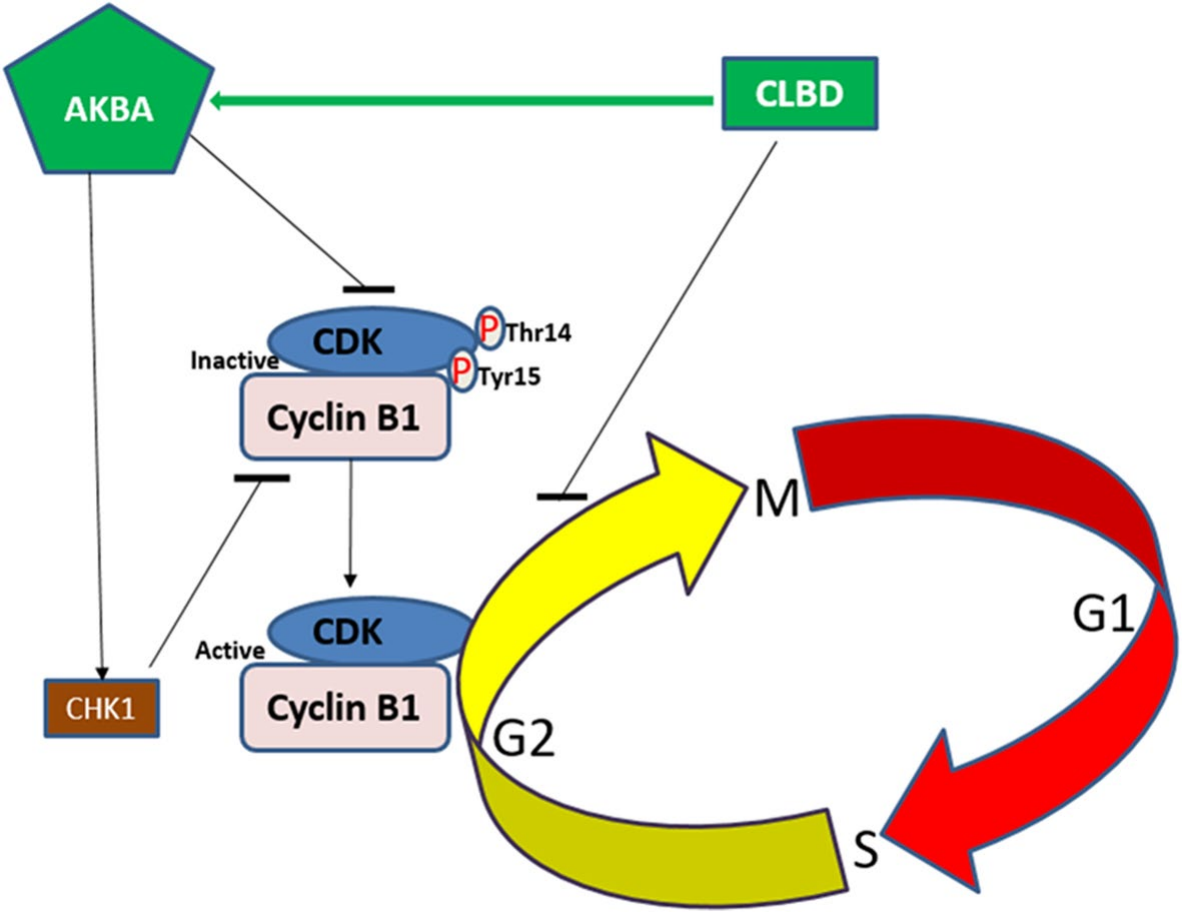
Blockage of cell cycle progression by chloroform extract and acetyl-11-keto-beta-boswellic acid obtained from *Boswellia dalzielii* stem bark at G2/M Phase. CLBD (chloroform extract of the Boswellia dalzielii stem bark)

The reverse virtual screening (rVS) of AKBA on some randomly selected cell cycle and/or apoptosis regulator protein targets revealed it is a multi-target compound, having the most favorable affinity for p-CDK1 within the constructed biomolecular library. Cyclin/CDKs complexes are the major controllers the cell cycle progression via phosphorylation of the target genes [19]. The data obtained in this study showed that AKBA, identified in the chloroform fraction of *Boswellia dalzielii* could have blocked the G2/M phase progression in AW8507 cells earlier reported [11], by downregulating the expression of CDK1 as it demonstrated a favorable affinity of − 9.2 kcal/mol with p-CDK1 in silico, whereas AKBA affinity for the Cyclin B1-CDK1 binding interface was comparatively low (-6.7 kcal/mol) which further indicated that AKBA directly inhibits CDK1 preferentially. According to the docking scores obtained, AKBA also demonstrated a good binding affinity with CHK1 protein (binding score =  − 8.5 kcal/mol), but unfavorable affinity with the active site of CDC25C with a positive energy of 230.9 kcal/mol. As shown in Fig. 6, the binding of AKBA preferably to p-CDK1 and at the same time to CHK1, collectively leads to the suppression of CDK1-cyclin B1 complex which has been found to be highly expressed in the more aggressive cancer phenotype [20], thus blocking the cell cycle progression.

Furthermore, the affinity of AKBA with EGFR and PI3K observed in silico (Table 1) further potentiates the efficacy of AKBA to block proliferation in head and neck cancer through multiple target proteins; however, more studies in vivo and/or in vitro would be required to confirm these results. In addition, the tendency of AKBA to bind β-Tubulin with a stronger binding affinity (− 8.9 kcal/mol) when compared to docetaxel (− 8.3 kcal/mol) (Table 2), further signified the possibility of compound AKBA being responsible for the apoptosis-induction potential of chloroform extract obtained from *Boswellia dalzielii* stem bark (CLBD) in AW8507 cells of the head and neck cancer because inhibition of β-Tubulin and CDK1 have been implicated in the obstruction of the anti-apoptotic proteins B-cell lymphoma extra-large (Bcl-XL) and B-cell lymphoma 2 (Bcl-2) [21, 22].

## Conclusions

Taking the obtained findings together, chloroform extract obtained from *Boswellia dalzielii* stem bark is an inducer of apoptosis in AW8507 cells, head, and neck cancer cell line. The reverse virtual docking of the compound acetyl-11-keto-beta-boswellic acid on selected proteins confirmed it possessed affinity for multiple protein targets in the pathways associated with the cell cycle, with highest affinity for phosphorylated-cyclin dependent kinase 1 (p-CDK1). Compound acetyl-11-keto-beta-boswellic acid also demonstrated higher affinity for αβ tubulin than the anti-head and neck cancer standard drug, docetaxel. Thus, acetyl-11-keto-beta-boswellic acid was responsible for the anticancer activity of *Boswellia dalzielii* stem bark extract earlier reported in vitro. Further in vitro and/or in vivo studies would be required to validate in silico observations, especially in head and neck squamous cell carcinoma.

## Data Availability

All materials and all data generated during this study are included in this research article.

## Abbreviations

EGFR: Epidermal growth factor receptor
αβ Tubulin: Alpha-beta tubulin
PI3K: Phosphoinositide-3-kinase
CHK1: Checkpoint kinase 1
CLBD: Chloroform extract of Boswellia dalzielii stem bark ethanol extract
TXL: Docetaxel
AKBA: Acetyl-11-keto-beta-boswellic acid
CDC25C: Cell Division Cycle 25 homolog C
CDK1: Cyclin dependent kinase 1
HNSCC: Head and neck squamous cell carcinoma
FOXM1: Forkhead box protein M1
PLK1: Serine/threonine polo-like kinase 1
Cyclin B1, TOP2A: DNA topoisomerase 2 alpha
Akt: Protein kinase B
HER2: Proto-oncogene Neu
c-MET: Mesenchymal epithelia transition factor
TP53: Tumor protein p53, MDM2mouse double minute 2 homolog protein
JAK: Janus tyrosine kinase
STAT3: Signal transducer and activator of transcription 3
MAPK: Mitogen activated protein kinase
VEGFR: Vascular endothelia growth factor receptor
